# Outlier Detection in Wireless Sensor Networks Using Model Selection-Based Support Vector Data Descriptions

**DOI:** 10.3390/s18124328

**Published:** 2018-12-07

**Authors:** Zhan Huan, Chang Wei, Guang-Hui Li

**Affiliations:** 1 School of Information Science & Engineering, Changzhou University, Changzhou 213164, China; hzh@cczu.edu.cn; 2 School of IoT Enginering, Jiangnan University, Wuxi 214122, China; weichang92@126.com; 3 Research Center of IoT Technology Application Engineering (MOE), Wuxi 214122, China

**Keywords:** outlier detection, wireless sensor networks, support vector data description, random feature mapping, model selection

## Abstract

Wireless sensor networks (WSNs) are often deployed in harsh and unattended environments, which may cause the generation of abnormal or low quality data. The inaccurate and unreliable sensor data may increase generation of false alarms and erroneous decisions, so it’s very important to detect outliers in sensor data efficiently and accurately to ensure sound scientific decision-making. In this paper, an outlier detection algorithm (TSVDD) using model selection-based support vector data description (SVDD) is proposed. Firstly, the Toeplitz matrix random feature mapping is used to reduce the time and space complexity of outlier detection. Secondly, a novel model selection strategy is realized to keep the algorithm stable under the low feature dimensions, this strategy can select a relatively optimal decision model and avoid both under-fitting and overfitting phenomena. The simulation results on SensorScope and IBRL datasets demonstrate that, TSVDD achieves higher accuracy and lower time complexity for outlier detection in WSNs compared with existing methods.

## 1. Introduction

WSNs are composed of a large number of sensor nodes, which are equipped with wireless transceivers, low-power microcontrollers, energy sources and various sensors [1]. A wide variety of applications of WSNs can be found, ranging from personal spaces to the scientific, industrial, business, and military domains. However, sensor observations collected from sensor nodes often have low data quality and reliability due to the limited capability of sensor nodes in terms of energy, memory, computational power, bandwidth, and the harshness of the deployment environment [2,3]. The use of low quality sensor data in any data analysis and decision-making process limits the possibilities for reliable and real-time situation-awareness. A solution to ensure the quality of sensor data is outlier detection. In addition, outlier detection can help diagnose the health condition of WSNs and identify the environmental events (such as forest fire, air pollution, etc.) [4,5,6]. Therefore, it is very important to find an effective and efficient outlier detection technique for WSNs, which should be able to identify outliers with high accuracy and a low false alarm rate, while satisfying the constraints in terms of memory and computational complexity [7].

In WSNs, outliers can be defined as ‘those measurements that significantly deviate from the normal pattern of sensed data’ [8]. In recent years, researchers have shown increased interest in applying machine learning approaches for outlier detection in WSNs. Zhang [9] classified outlier detection techniques into statistical-based, nearest neighbor-based, clustering-based, classification-based, spectral decomposition-based and other types. SVDD is a one-class classification technique, its main idea is to find a minimum hyper-sphere such that all or most acceptable data samples are enclosed in the hyper-sphere [10,11]. The boundary of the hyper-sphere is the decision boundary, which can be used to identify outlier data. To reduce the high computational complexity of SVDD, Platt [12] and Fan [13] proposed sequential minimal optimization (SMO) algorithms to calculate the quadratic optimization required in the SVDD algorithm, effectively reducing the time complexity to O(*n*^2^). Chang [14] proposed an SMO optimization method for resolving dual quadratic optimization problems by using decomposition methods. Liu [15] and Feng [16] proposed to directly find a hyper-sphere or hyper-ellipsoid preimage of the feature vector, and then used a simple relationship between this feature vector and the SVDD sphere center to re-express the center with a single vector. Although the above-mentioned methods can reduce the computational complexity of the SVDD, none of them solves the problem of excessive calculation required for kernel functions. In 2007, Rahimi [17,18] mapped the input data to a randomized low-dimensional feature space and then applied existing fast linear learning methods. A significant speed up can be achieved by computing random features. However, it needs to maintain a high accuracy when the feature space dimension is high enough. Sutherland [19] improved the uniform error bound of paper [17], as well as gave a novel understanding of the embedding’s variance, approximation error, and use in some machine learning methods. Aman [20] extends the randomized-feature approach to the task of learning a kernel (via its associated random features), and presents an efficient optimization problem that learns a kernel in a supervised manner. They proved the consistency of the estimated kernel as well as generalization bounds for the class of estimators induced by the optimized kernel. Andrea [21] proposed an explicit description of the reproducing kernel Hilbert space (RKHS) induced by the approximated Gaussian kernel. They demonstrated that the approximations had indistinguishable performance from the full kernels yet greatly reduce the train/test times of SVMs.

The majority of existing outlier detection methods for WSNs does not take into account multi- dimensional data and assume the sensor data is univariate. They ignore the fact that the attributes together can display anomaly while in some cases none of the attributes individually has an anomalous value. However, each sensor node may be equipped with multiple sensors and also certain correlations may exist among attributes of sensor data. The attributes together can display anomaly while in some cases none of the attributes individually has an anomalous value. Thus, outlier detection techniques for WSNs should be able to analyze multi-dimensional data and identify whether the attributes together display anomaly [9].

For outlier detection in WSNs, it is necessary to maintain a high accuracy at a low feature space dimension. To meet this goal, this paper proposes an outlier detection method (TSVDD) using model selection-based SVDD, and this method can analyze multi-dimensional sensor data and improve the accuracy of outlier detection. The novelty of this method lies in the model selection strategy, which can help select a relatively optimal decision model and avoid both under-fitting and overfitting phenomena. The remainder of the paper is organized as follows: First we introduce the basic idea of SVDD and the random Fourier feature in Section 2. In Section 3 we discuss the proposed outlier detection method (TSVDD). Experimental results will be shown in Section 4, and we provide our conclusions in Section 5.

## 2. Support Vector Data Description and Random Fourier Feature

In this section, we introduce the principle behind the SVDD algorithm and the random Fourier feature, which are related to our research.

### 2.1. Support Vector Data Description

**Figure 1:**
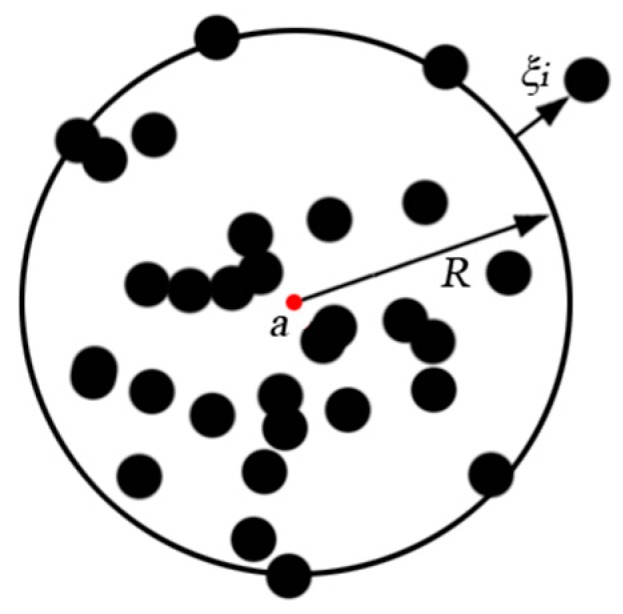
Geometry model of SVDD.

Given a target training set with *n* data objects, the main idea of SVDD [10,11] is to find a minimum-volume sphere with center *a* and radius *R* such that all or most of the target training data can be enclosed by the sphere. The optimization problem can be formulated as:

(1) $$\begin{array}{l} \underset{R}{\min}\;R^{2}+C\sum_{i=1}^{n}\xi_{i}\\ s.t.\;(x_{i}-a){\left(x_{i}-a\right)}^{T}\leq R^{2}+\xi_{i} \end{array}$$

where *ξ_i_* are the slack variables, *C* is the penalty weight, which gives the trade-off between the two error terms: volume of the sphere and the number of target objects rejected. The geometric model of SVDD is shown in Figure 1. The black points in Figure 1 are the data objects.

Incorporating the constraints in Equation (1), we construct the Lagrangian:

(2) $$L(R,a,\alpha_{i},\xi_{i})=R^{2}+C\sum_{i=1}^{n}\xi_{i}-\sum_{i=1}^{n}\alpha_{i}(R^{2}+\xi_{i}-(x_{i}^{2}-2ax_{i}+a^{2}))-\sum_{i=1}^{n}\gamma_{i}\xi_{i}$$

Using Lagrange multipliers *α_i_* ≥ 0 and *γ_i_* ≥ 0, setting ∂L/∂R=0, ∂L/∂a=0, and ∂L/∂i=0, and then substituting the results back into Equation (2), we obtain the dual problem:

(3) $$L=\sum_{i=1}^{n}\alpha_{i}(x_{i}\cdot x_{i})-\sum_{i=1}^{n}\sum_{j=1}^{n}\alpha_{i}\alpha_{j}(x_{i}\cdot x_{j})$$

However, this method only has good performance for data sets whose input space is spherically distributed. To find a more flexible method, the data objects can be transformed into a higher dimensional feature space. The inner products in Equation (3) can be replaced by a kernel function *K* (*x_i_*, *x_j_*). Here, we select the radial basis function as the kernel function:

(4) $$K(x_{i},x_{j})=\phi (x_{i})\cdot \phi (x_{j})=\exp(\frac{-{\left(x_{i}-x_{j}\right)}^{2}}{2\delta^{2}})$$

For this Gaussian kernel function *K* (*x_i_*, *x_i_*) ≡ 1, hence Equation (3) can be transformed as:

(5) $$W=\underset{\alpha }{\max}\overset{n}{\underset{i=1}{\sum }}\overset{n}{\underset{j=1}{\sum }}\alpha_{i}\alpha_{j}K\left(x_{i},x_{j}\right)-1\;s.t.\sum_{i=1}^{n}\alpha_{i}=1,\;0\leq \alpha_{i}\leq C\left(\forall i=1,\;2,\ldots ,n\right)$$

Equation (5) is a typical quadratic optimization problem, the target training data can be classified into the three categories: (i) *α_i_* = 0, the data that are inside the sphere; (ii) 0 < *α_i_* < *C*, the data that are on the boundary of the sphere; and (iii) *α_i_* = *C*, the data that are outside the sphere. Hence, we have the SVDD decision function:

(6) $$f(x_{i})=\mathrm{sgn}(||\phi (x_{i})-a|{|}^{2}-R^{2})$$

if *f*(*x_i_*) = −1, *x_i_* is accepted as a target data; otherwise *x_i_* is labeled as an outlier data.

### 2.2 Random Fourier Feature

**Figure 2:**
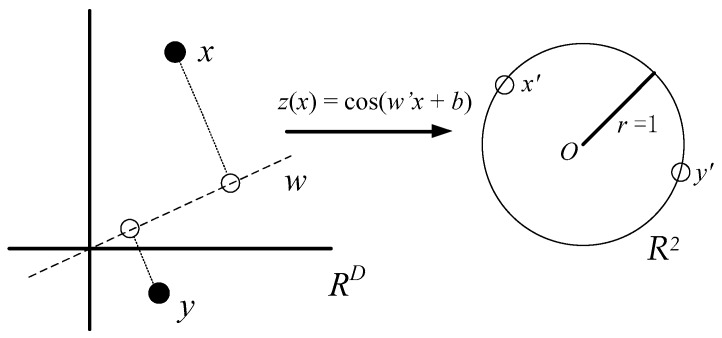
Random Fourier Feature map.

The first set of random features consists of random Fourier bases cos(*ω*′*x* + *b*) where *ω* ∈ *R^D^* and *b* ∈ *R* are random variables. These mappings first project data points on a randomly chosen line, and then pass the resulting scalar through a sinusoidal function (see Figure 2). Drawing the direction of these lines from an appropriate distribution guarantees that the product of two transformed points will approximate a desired shift-invariant kernel [17].

For example, in Figure 2, each component of the feature map *z*(*x*) projects *x* and *y* onto a random direction *ω* drawn from the Fourier transform *p*(*ω*) of *k*(Δ), and wraps this line onto the unit circle in *R*^2^. After transforming two points *x* and *y* in this way, their inner product is an unbiased estimator of *k*(*x*, *y*) [17]. The map *z*(*x*) = cos(*ω’x* + *b*) additionally rotates this circle by a random amount *b* and projects the points onto the interval [0, 1].

**Theorem** **1.** *(Bochner [22]) A continuous kernel k(x,y) = k(x − y) on R^D^ is positive definite if and only if k(δ) is the Fourier transform of a non-negative measure. If k(δ) is properly scaled, Bochner’s theorem guarantees that its Fourier transform p(w) is a proper probability distribution [17]*.

*Defining $\varsigma_{w}\left(x\right)=e^{jw^{\prime }x}$, we have:*

(8) $$k(x-y)=\int_{R^{d}}p(w)e^{jw^{’}(x-y)}d_{w}=E_{w}[\varsigma_{w}(x)\varsigma_{w}{\left(y\right)}^{*}]$$

From Equation (8), we know that $\varsigma_{w}{\left(x\right)}_{w}{\left(y\right)}^{*}$ is an unbiased estimator of *k*(*x*,*y*) when *w* is drawn from *p*(*w*). We can lower the variance of $\varsigma_{w}\left(x\right)\varsigma_{w}{\left(y\right)}^{*}$ by concatenating *D* randomly chosen *ς_w_* into a column vector *ς* and normalizing each component by $\sqrt{D}$. The inner product of points characterized by the 2D-dimensional random feature *ς*, $\varsigma_{w}\left(x\right)\varsigma_{w}{\left(y\right)}^{*}$ = 1D∑j=1Dςwj(x)ςwj(y)* is a sample average of $\varsigma_{wj}\left(x\right)\varsigma_{wj}\left(y\right)$, and is therefore a lower variance approximation to the expectation (8) [17].

## 3. Outlier Detection Algorithm Using Model Selection Based Support Vector Data Description

SVDD is an excellent one-class classification algorithm. However, kernel function calculation is complex. This paper proposes an outlier detection algorithm using model selection-based SVDD (TSVDD), which can reduce the computational complexity, while maintaining high detection accuracy. TSVDD consists of random Fourier feature mapping and model selection.

### 3.1. Toeplitz Random Fourier Feature Mapping in Support Vector Data Description (TRFF)

In WSNs, the distribution of sensor data is often irregular. The linear SVDD algorithm is not suitable for outlier detection. Therefore, this paper chooses the kernel-based SVDD, and the radial basis function is used as the kernel function. Considering the resource limitation of sensor nodes, we apply the Toeplitz random Fourier feature mapping to reduce the computational complexity of kernel function-based SVDD algorithm (kernel_SVDD).

**Definition** **1.** *A Toeplitz matrix is a matrix in which each descending diagonal from left to right is constant. For instance, matrix T given in Equation (9) is a Toeplitz matrix*.

(9) $$T=\left[\begin{array}{cccc} a & b & c & d\\ e & a & b & c\\ f & e & a & b \end{array}\right]$$

A circulant matrix is a special kind of Toeplitz matrix, where each row vector is rotated one element to the right relative to the preceding row vector. Equation (10) shows an instance:

(10) $$T=\left[\begin{array}{ccc} a & f & e\\ e & a & f\\ f & e & a \end{array}\right]$$

**Lemma** **1.** *Suppose the D-dimensional vector T(1)~N(0, I_D_/δ^2^), and the circulant matrix T_D_ is constructed by Toeplitz transform using T(1). Then T_D_ satisfies the following condition:*

(11) $$E\left(\varphi \left(x_{i}\right)\varphi {\left(x_{j}\right)}^{\prime }\right)=k\left(x_{i},x_{j}\right)$$

*where, $\varphi \left(x_{i}\right)=\frac{1}{\sqrt{D}}e^{iT_{D}x_{i}}$, $k\left(x_{i},y_{j}\right)=exp\left(-\frac{{∥x_{i}-y_{j}∥}^{2}}{2\delta^{2}}\right)$*.

Therefore, the algorithm only needs to store the first column vector so that we can reconstruct the whole matrix, and the space complexity is only *O*(*n*), so we use the Toeplitz random matrix to substitute the random Fourier feature matrix *W* for random feature mapping, and reduce the computational complexity of the SVDD algorithm. Actually, SVDD typically suffers from cubic complexity since it needs to solve convex quadratic programming problems, and it’s very difficult to be used directly in resource-constrained WSNs. Using the Toeplitz random Fourier feature to approximate the radial basis function, we propose the Toeplitz random Fourier feature SVDD algorithm (TRFF), which is described as follows.

*Step 1*: Initialize the radial basis function parameter *δ* and the feature dimension *D*.
*Step 2*: Draw samples *T*(1) from *N* (0, *I_D_*/*δ*^2^);
*Step 3*: Use the Toeplitz transformation to obtain the *D*-dimensional matrix *T_D_*;
*Step 4*: Compute the approximate radial basis function KM_RFF by Equation(11);
*Step 5*: Solve the QP problem using the SMO algorithm for KM_RFF;
*Step 6*: Construct the decision function $f\left(x\right)=sgn\left({∥e^{iT_{D}}-a∥}^{2}-R^{2}\right)$ of the TRFF algorithm.

### 3.2. Model Selection

The traditional random feature mapping algorithm has poor stability in low dimensional feature spaces. Thus, the phenomenon of over-fitting or under-fitting often happens in the decision model. In this study, we combine the model selection strategy with the TRFF algorithm to avoid the over-fitting and under-fitting in the SVDD.

**Claim** **1.** *Uniform convergence of Fourier features [17]:*

(12) $$P[(\varsigma_{w}(x)\varsigma_{w}{\left(y\right)}^{*}-k(x,y))>\varepsilon ]\leq \exp(-2\varepsilon^{2}D^{2})$$

**Proof.**  Suppose *x*_1_, *x*_2_, …, *x_n_* are independent random variables, $a_{i}\leq x_{i}\leq b_{i}$, $\bar{x}=\frac{x_{1}+x_{2}+\cdots +x_{n}}{n}$, then for any *δ* > 0, Hoeffding’s inequality provides an upper bound on the probability that the sum of bounded independent random variables deviates from its expected value by more than a certain amount: $P\left\{\bar{X}-E\left(X\right)\geq \delta \right\}\leq \exp\left(-2\delta^{2}N^{2}\right)$. Since $\varsigma_{w}\left(x\right)\varsigma_{w}{\left(y\right)}^{*}$ is an unbiased estimator of *k*(*x*,*y*), and $E[\varsigma_{w}(x)\varsigma_{w}{\left(y\right)}^{*}]=k(x,y)$, we can conclude that $P[(\varsigma_{w}(x)\varsigma_{w}{\left(y\right)}^{*}-k(x,y))>\varepsilon ]\leq \exp(-2\varepsilon^{2}D^{2})$. □

If the error between the radial basis function and its unbiased estimator is *ε* = 0.1, and the random feature mapping dimension is *D* = 10, then $P[(\varsigma_{w}(x)\varsigma_{w}{\left(y\right)}^{*}-k(x,y))>0.1]\leq 0.1353$, hence the confidence level is 86.47%. If the error between the radial basis function and its unbiased estimator is *ε* = 0.01, and the random feature mapping dimension is *D* = 10, then:

(13) $$P[(\varsigma_{w}(x)\varsigma_{w}{\left(y\right)}^{*}-k(x,y))>0.01]\leq 0.9801$$

Hence, the confidence level is 1.99%. Given the error bound *ε* and random feature dimension *D*, the approximate random Fourier feature mapping with a difference from *k*(*x*,*y*) less than *ε* can be found at the confidence level. This proves that when the random feature dimension *D* is low, there is a relatively optimal unbiased estimator that approximates the kernel function. Therefore, the goal of model selection is to select a relatively optimal model.

#### 3.2.1 Under-Fitting Error

**Figure 3:**
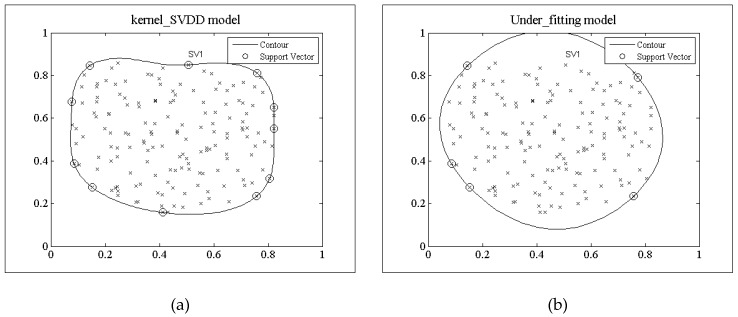
Under-fitting model (**a**) Model trained by kernel_SVDD; (**b**) Model trained by TRFF.

**Definition** **2.** *An under-fitted model is a model that cannot adequately capture the underlying structure of the data, where some parameters or terms that would appear in a correctly specified model are missing. The under-fitting model is shown as follow in Figure 3b*.

Figure 3 shows the decision model trained by the two algorithms under the random data set with 181 data objects generated by the Gaussian function *N*(0,1), where, all the data are 2D-dimentional, 160 data objects are normal and 21 data objects are outliers. In addition, the dimension of the random feature space is *D* = 10. Figure 3a shows the trained decision model of the kernel_SVDD algorithm. The support vector points accurately reflect the training dataset region. The contour line is the hyper-sphere in the feature space. Figure 3b is the trained decision model of TRFF algorithm under the random feature dimension *D* = 10. There is a mismatch between the model and the training dataset region, which does not reflect the characteristics of the region where the training dataset is located. If this model is used for outlier detection, it will inevitably lead to false positive alarms. Contrasting Figure 3a,b, there exists a big difference between the support vectors of the kernel_SVDD and the TRFF decision model.

#### 3.2.2 Over-Fitting Error

**Figure 4:**
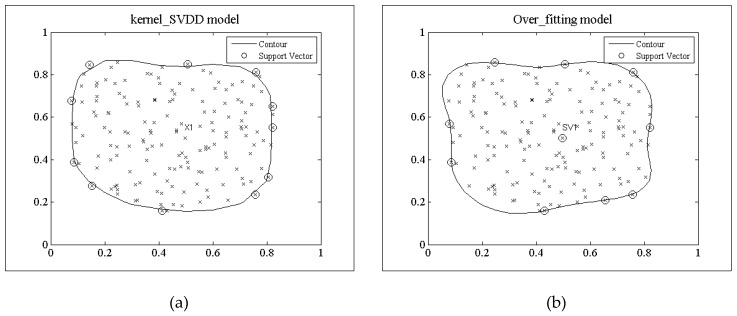
Over-fitted model (**a**) Trained model of kernel_SVDD algorithm; (**b**) Trained model of TRFF algorithm.

**Definition** **3.** *The over-fitted model: The production of a model that corresponds too closely or exactly to a particular set of data, and may therefore fail to fit additional data or predict future observations reliably. The over-fitting model is shown in Figure 4b*.

Figure 4 shows the decision models trained by the two algorithms using the same random data set used in Figure 3. Figure 4a gives the decision model of kernel_SVDD, and Figure 4b shows the decision model of TRFF trained under the random feature mapping when the dimensionality is *D* = 10. Its outer boundary is basically the same as that of the kernel_SVDD decision model. Hence, it correctly displays the training dataset region. The outer support vector point is also basically consistent with the kernel_SVDD. However, there have some internal support vector points shown in Figure 4b, which reflect that the data samples surrounding the internal support vector points are outliers, so this model will also lead to false negative alarms during the detection process as the under-fitting model. Model selection needs to avoid the internal support vector points.

To design an optimal decision model, we should avoid both over-fitting and under-fitting. Algorithm 1 gives the pseudocode of the model selection strategy for kernel_SVDD, where *T*(1) is a column vector drawn from a Gaussian distribution, *T_D_* is a *D*-dimensional random feature matrix from the Toeplitz transformation, and *error_under_τ_* is the given threshold of under-fitting error.**Algorithm 1** Model selection for Kernel_SVDDInput: Training dataset *Train* = {*x*_1_, *x*_2_, …, *x_n_*}   Support vector *SV_S_* of kernel_SVDDProcess:1: while (1) do2: Sample *T*(1)~*N*(0, *I_D_*/*δ*^2^);3: Apply the Toeplitz transformation of *T*(1) to form a *D*-dimensional feature matrix *T_D_*;4: Train the training set *Train* to obtain decision model *TRFF_f* using TRFF algorithm;5: Calculate the over-fitting error: *error_over*6: if *error_over* = 07:   Calculate the under-fitting error *error_under*;8:   if error_under < error_under_τ_9:    break;10:   else11:    continue;12:   end if;13: else14:  continue;15: end if;16: end while;Output: Random feature matrix of optimal model *T_D_*

Once the optimal model is derived, we can detect the outliers using this optimal decision model and SVDD. The proposed outlier detection algorithm (TSVDD) is shown in Algorithm 2.**Algorithm 2** TSVDD algorithmInput: Training dataset *Train*   Testing dataset: *Test* = {*x*_1_, *x*_2_, …, *x_n_*}Process:1: Derive the decision model *f* of SVDD use training dataset;2: While (*Test* ≠ *φ*) do3:  if *f*(*x_i_*) > 0, (*i* = 1, 2, …, *n*)4:   *x_i_* is marked as an outlier;5:  else6:   *x_i_* is marked as an adequate sample;7:  end if;8: end while;Output: the outlier set

## 4. Experimental Results

To evaluate the performance of the proposed outlier detection algorithm, we carried out several simulation experiments on two WSN data sets, and compared the results of TSVDD with those of FastFood [23], RFF [17] and the traditional kernel_SVDD. All algorithms are implemented using Matlab 2014a, in a PC with equipped with an Intel (R) corei3 dual-core 3.6 GHz CPU, 4 G memory, and the Windows 7 operating system.

### 4.1. Data Sets

#### 4.1.1. IBRL Dataset

The IBRL dataset was collected from 54 sensors deployed in the Intel Berkeley Research lab between February 28th and April 5th, 2004. Mica2Dot sensors with weather boards collected time stamped topology information, along with humidity, temperature, light and voltage values once every 31 s. Data was collected using the TinyDB in-network query processing system, built on the TinyOS platform. Considering the data integrity and continuity, we chose the humidity data and temperature data from Node 51 as the experimental dataset. We use IBRL_51 to represent this dataset. It is notable that there are some missing epochs in IBRL.

#### 4.1.2. SensorScope Dataset

The SensorScope system [24] was deployed at the Grand-St-Bernard pass at 2400 m between Switzerland and Italy. The SensorScope dataset was collected from 13 September 2007 to 26 October 2007 and includes ambient temperature, surface temperature and relative humidity data. The sampling period was 2 min. Similarly, considering the data integrity and continuity, we selected the ambient temperature data and surface temperature data from Node 12 as the experimental dataset. We use SS_12 to represent this data set. Similarly, there are some missing data in SS_12.

Table 1 lists the abovementioned datasets used for our experiments. To evaluate the robustness of the proposed algorithm, we chose three sub-datasets (numbered 1, 2, 3, respectively) from different periods for each sensor. For example, IBRL_51-1, IBRL_51-2 and IBRL_51-3 consisted of the sensor data of 2 days, 4 days and 5 days, respectively, SS_12_1, SS_12_2, and SS_12_3 consisted of the sensor data of 3 days, 6 days and 9 days, respectively, and then we divided them into training sets and testing sets with almost the same ratio. By preliminarily statistical analysis, we found that the abnormal data account for 8% to15% of the testing datasets.

### 4.2. Performance Metrics

For the one-class classification problem, the samples can be classified into four types: true positive *(TP*), false positive (*FP*), true negative (*TN*) and false negative (*FN*), according to the combination of their true category and decided category by the algorithms, which can be described by a confusion matrix [20], as shown in Table 2.

The following four metrics can be calculated from the confusion matrix: True Positive Rate (*TPR*), True Negative Rate (*TNR*), False Positive Rate (*FPR*), and False Negative Rate (*FNR*). The calculation formulas are as follows:

(13) $$TPR=\frac{TP}{TP+FN}$$

(14) $$FPR=\frac{FP}{FP+TN}$$

Since *TPR* + *FNR* = 1, *TNR* + *FPR* = 1, we only use *TPR* and *FPR* as the performance evaluation metrics in this paper.

### 4.3 Performance Comparison Among Different Outlier Detection Algorithms

In our experiments, we set the radial basis function parameter *δ* = 0.8, and the regularization parameter is *C* = 1. Considering the randomness, we finished 100 runs of each algorithm for outlier detection, and then compared the average results. Table 3 shows the average and standard deviations of *TPR* and *FPR* of 100 experiments for five different algorithms, where RFF_30 and RFF_300 represent the RFF algorithm [17] with random feature dimension *D* = 30 and *D* = 300 respectively, TSVDD is the proposed algorithm in this study, and FastFood is the algorithm given in [23]. Because the kernel_SVDD has no random feature, its experimental results are unchanged, then we used these results as the references for comparison. The bold number represents the smallest standard deviation under the current data set. The smaller standard deviation reflects the more stable algorithm. Table 3a,b give the results of under the SS_12 dataset. It can be seen that, for FastFood and RFF_30, their average values of *TPR* and *FPR* are relatively high compared with the other algorithms. For RFF_300 and TSVDD, the standard deviation is very low, but *TPR* and *FPR* are very close to those of kernel_SVDD algorithm. It shows that RFF_300 and TSVDD completed the random feature mapping to approximate the kernel function precisely. Table 3c,d show the results under IBRL_51 dataset. Comparatively, RFF_300 and TSVDD have lower standard deviation values, so their results are more stable. In the case of low random feature dimension, TSVDD has almost the same *FPR* and *TPR* as kernel_SVDD.

**Figure 5:**
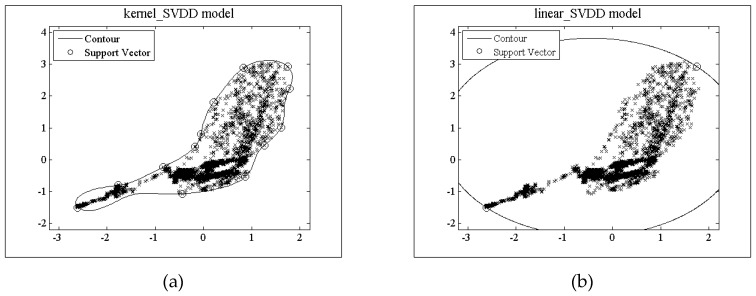

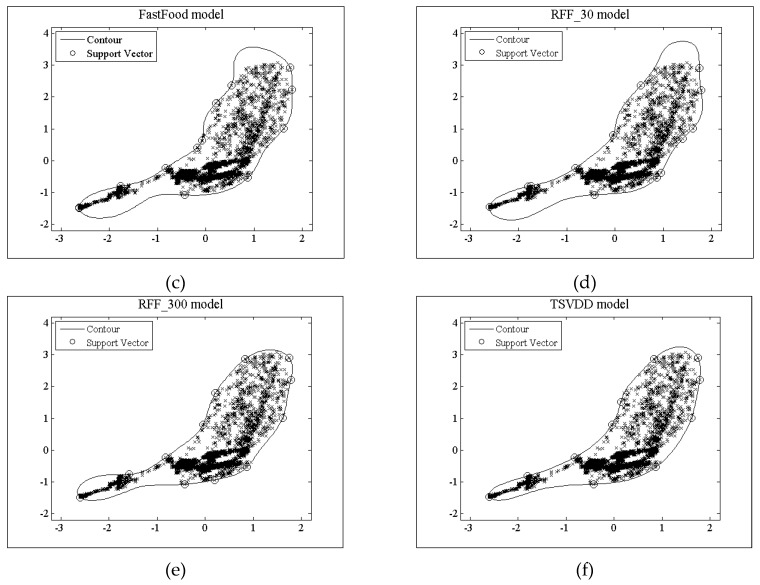
Decision models trained by different algorithm on SS_12-3 dataset (**a**) kernel_SVDD model; (**b**) linear_SVDD model; (**c**) FastFood model; (**d**) RFF_30 model; (**e**) RFF_300 model; (**f**) TSVDD model.

Figure 5 shows the training decision models of kernel_SVDD, linear_SVDD, FastFood, RFF and TSVDD on SS_12-3 dataset. Figure 5a gives the model trained by kernel_SVDD, which accurately describes the edge of the data. The models represented by Figure 5e,f are trained by RFF_300 and TSVDD, both models are similar with that of kernel_SVDD, and their data edges are basically accurate. From Figure 5c,d, it can be seen that the models trained by FastFood and RFF_30 are under-fitting models, which will inevitably lead to false positives.

**Figure 6:**
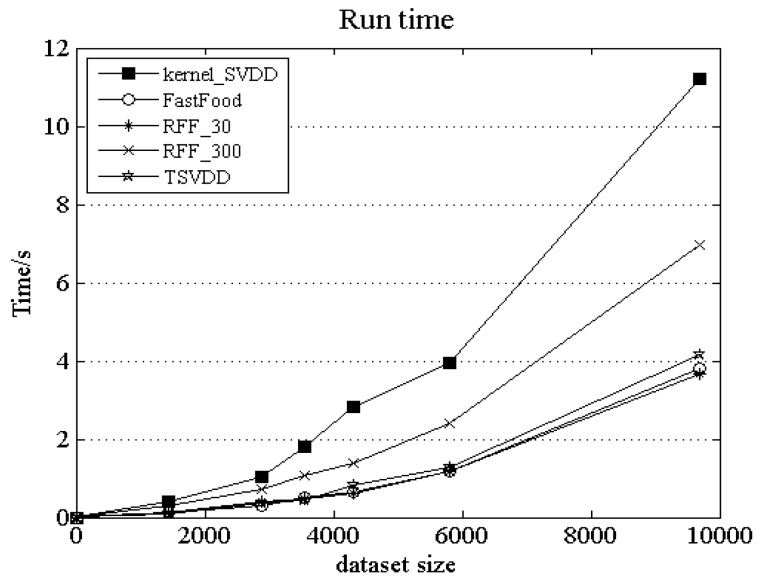
Comparison of run time.

Figure 6 presents the run time for outlier detection on the given six datasets, which does not include the model training time. Here, the data size is the sample number of the testing dataset. From Figure 6, we can see that the run time of TSVDD on each dataset is not more than that of the other algorithms. For large datasets, the run times of kernel_SVDD and RFF_30 are longer than the other algorithms. For IBRL_51-3 dataset, its data size is 9694, and the run time of kernel_SVDD is almost three times that of TSVDD. When the data size increases, the time difference between RFF_300 and TSVDD will become increasingly greater. Due to the higher dimensionality of RFF_300 random feature mapping, the dataset size has a greater impact on its run time, so TSVDD has the highest efficiency while maintaining a high detection accuracy compared with the other algorithms.

**Figure 7:**
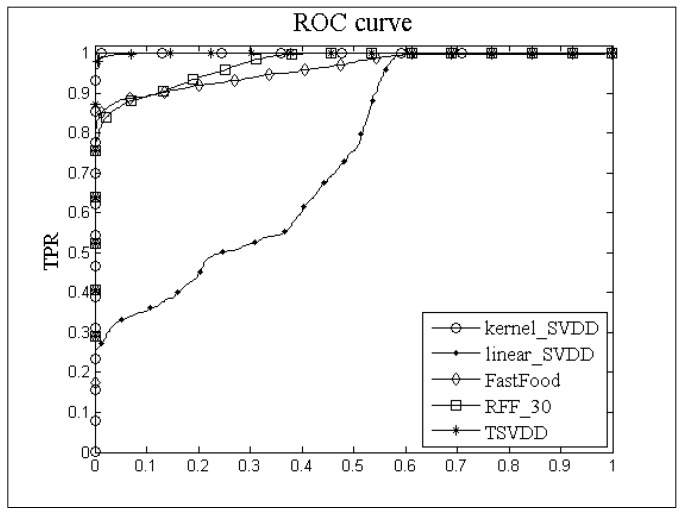
ROC curve.

The Receiver Operating Characteristic (ROC) curve is often applied to judge the performance of outlier detection algorithms, the area under the ROC curve is called Area under ROC Curve (AUC). Figure 7 shows the ROC curves of kernel_SVDD, linear_SVDD, FastFood, RFF, and TSVDD for the IBRL_51-3 dataset. From Figure 7 it can be seen that TPR of TSVDD is better than FastFood and RFF for the same random feature dimensions. Meanwhile, the TPR values of TSVDD and kernel_SVDD are almost equal to 1, so TSVDD is very accurate for outlier detection. From our experiments, we found that the ROC curves have the same trends for all other datasets. Generally, in low random feature dimensional cases, TSVDD has a higher *TPR* and a lower *FPR*, and it can remain maintain a stable performance for different datasets. Compared with kernel_SVDD and high dimensional random feature mapping algorithms, TSVDD has higher efficiency.

## 5. Conclusions

This study proposes an outlier detection algorithm (TSVDD) for wireless sensor networks. TSVDD aims to solve two issues of traditional SVDD algorithms. The first issue is the high computational cost of radial basis function calculations. To reduce the computational complexity, a Toeplitz random feature mapping with circulant matrix projection is used for approximating the radial basis function. The second issue is the poor stability of the traditional random feature mapping in low dimension feature space. A model selection strategy for avoiding over-fitting and under-fitting errors is proposed to guarantee the stability at low random feature dimensions. Simulation results on different datasets show that TSVDD has higher detection accuracy, a lower false alarm rate and higher efficiency than other traditional algorithms.

## Figures and Tables

**Table 1:** Experimental datasets.

Dataset Source	Data Type	Dataset Number	Size of Training Dataset	Size of Testing Dataset
SensorScope	Temperature & humidity	SS_12-1	717	1473
		SS_12-2	1440	2877
		SS-12-3	2157	4303
IBRL	ambient temperature & surface temperature	IBRL_51-1	1822	3562
		IBRL_51-2	3562	5816
		IBRL_51-3	5068	9694

**Table 2:** Confusion matrix of classification results.

True category	Decision category	
	Outlier	Normal
Outlier	*TP*	*FN*
Normal	*FP*	*TN*

**Table 3:** Performance comparison of various algorithms under different data sets.

(**a**) *TPR* comparison of SS_12 dataset.									
**Data Set**	**Kernel_SVDD (%)**	**FastFood**		**RFF_30**		**RFF_300**		**TSVDD**	
		**Avg (%)**	**std**	**Avg (%)**	**std**	**Avg (%)**	**std**	**Avg (%)**	**std**
SS_12-1	99.79	99.77	0.0022	99.78	0.0033	99.71	0.0022	99.82	0.0013
SS_12-2	99.21	97.84	0.0144	98.29	0.0129	98.68	0.0082	98.55	0.0112
SS_12-3	96.82	96.87	0.0191	96.92	0.0153	97.32	0.0064	96.58	0.0086
(**b**) *FPR* comparison of SS_12 dataset									
**Data Set**	**Kernel_SVDD (%)**	**FastFood**		**RFF_30**		**RFF_300**		**TSVDD**	
		**Avg (%)**	**std**	**Avg (%)**	**std**	**Avg (%)**	**std**	**Avg (%)**	**std**
SS_12--1	10.81	28.22	0.1427	27.30	0.1428	14.32	0.0361	16.76	0.0444
SS_12-2	0.00	1.62	0.0139	1.21	0.0122	0.12	0.0016	0.72	0.0031
SS_12-3	0.75	8.80	0.0868	7.26	0.0879	1.28	0.0104	1.46	0.0108
(**c**) *TPR* comparison of IBRL_51 dataset									
**Data Set**	**Kernel_SVDD (%)**	**FastFood**		**RFF_30**		**RFF_300**		**TSVDD**	
		**Avg (%)**	**Std**	**Avg (%)**	**Std**	**Avg (%)**	**Std**	**Avg (%)**	**Std**
IBRL_51-1	99.76	97.58	0.0364	97.42	0.0323	99.14	0.0116	99.28	0.0066
IBRL_51-2	99.43	98.15	0.0152	97.92	0.0152	98.19	0.0115	97.96	0.0019
IBRL_51-3	99.73	99.26	0.0092	99.49	0.0062	99.49	0.0044	99.37	0.0090
(**d**) *FPR* comparison of IBRL_51 dataset									
**Data Set**	**Kernel_SVDD (%)**	**FastFood**		**RFF_30**		**RFF_300**		**TSVDD**	
		**Avg (%)**	**Std**	**Avg (%)**	**Std**	**Avg (%)**	**Std**	**Avg (%)**	**Std**
IBRL_51-1	0.55	3.61	0.0448	2.61	0.0319	1.89	0.0147	1.88	0.0236
IBRL_51-2	0.06	2.72	0.0224	2.27	0.0213	0.68	0.0085	0.41	0.0022
IBRL_51-3	0.25	4.78	0.0352	5.87	0.0411	1.28	0.0156	1.14	0.0046
